# DNA Damage Induces Down-Regulation of Prp19 via Impairing Prp19 Stability in Hepatocellular Carcinoma Cells

**DOI:** 10.1371/journal.pone.0089976

**Published:** 2014-02-28

**Authors:** Jie Yin, Yi-An Zhang, Tao-Tao Liu, Ji-Min Zhu, Xi-Zhong Shen

**Affiliations:** Department of Gastroenterology, Zhongshan Hospital of Fudan University, Shanghai, China; University of North Carolina School of Medicine, United States of America

## Abstract

Pre-mRNA processing factor 19 (Prp19) activates pre-mRNA spliceosome and also mediates DNA damage response. Prp19 overexpression in cells with functional p53 leads to decreased apoptosis and increases cell survival after DNA damage. Here we showed that in hepatocellular carcinoma (HCC) cells with inactive p53 or functional p53, Prp19 was down-regulated due to the impaired stability under chemotherapeutic drug treatment. Silencing Prp19 expression enhanced apoptosis of HCC cells with or without chemotherapeutic drug treatment. Furthermore high level of Prp19 may inhibit chemotherapeutic drugs induced apoptosis in hepatocellular carcinoma cells through modulating myeloid leukemia cell differentiation 1 expression. These results indicated that targeting Prp19 may potentiate pro-apoptotic effect of chemotherapeutic agents on HCC.

## Introduction

Hepatocellular carcinoma (HCC) is the fifth most common cancer and the fourth leading cause of cancer-related mortality worldwide [1]. Although surveillance in high-risk population allows diagnosis of HCC at early stage, most cases still present at advanced and unresectable stage, and limited therapeutic options are available [2]. The gloomy prognosis of the disease is also attributed to inefficiency of traditional chemotherapeutic agents.

Apoptosis, an important physiological process of cell death, occurs during tissue remodeling, immune regulation, and tumor regression. Generally, chemotherapeutic drugs induce the death of apoptotic cell in a mitochondrial pathway by activating BH3-only proteins and neutralizing the anti-apoptotic proteins such as B-cell lymphoma (BCL) 2, BCL-extra large (BCL-xL) and myeloid leukemia cell differentiation 1 (MCL1) [3]. Activities of such anti-apoptotic proteins are orchestrated by some tumor suppressors including p53 [4], which also plays key roles in apoptosis. However mutation in p53 barely presents in approximately 20–45% of HCC [5], indicating that p53-independent mechanisms are probably involved in chemotherapeutic-mediated apoptosis in HCC. Thus exploring the unknown apoptotic regulator could offer more insight into the chemotherapeutic resistance of HCC.

As an essential pre-mRNA splicing factor, pre-mRNA processing factor 19 (Prp19) plays a direct role in cellular response to DNA damage including repairing DNA damage, repressing cell cycle arrest and inhibiting apoptosis [6]. Depletion of Prp19 in HeLa cells results in accumulation of cellular apoptosis [7], whereas overexpression of Prp19 in HeLa cells can provide a pro-survival effect on DNA damage [8]. Therefore up-regulation of Prp19 may prolong human lifespan via reinforcing capacity of DNA damage repair or resistance to stress [9], [10]. In regard of its significant role in DNA damage repair and increased expression in colon and larynx cancer [11], it is reasonably argued that Prp19 is involved in development of cancer. Recent works also indicate that Prp19-associated complex protects cells from irradiation-induced apoptosis via inhibiting p53 mRNA expression or transcriptional activity [12], [13], function of Prp19 in HCC cells with mutated p53 remain, however, poorly understood. Therefore, we propose that Prp19 may be required for HCC cells to antagonize chemotherapeutic agents-induced apoptosis.

## Materials and Methods

### Cell Culture and Chemical Agents

Liver cancer cell lines (Huh7 and SMMC-7721) were purchased from Cell Bank of Type Culture Collection of Chinese Academy of Sciences (Shanghai, China). Cells were cultured in Dulbecco's modified Eagle's medium (Jinuo Biotec, Shanghai, china) with 10% fetal calf serum and a humidified incubator at 37°C in the presence of 5% CO_2_. Doxorubicin (Dox) and cisplatin (CDDP) are obtained from Sigma (Sigma-Aldrich, St. Louis, USA). Cycloheximide (CHX) is purchased from Beyotime (Beyotime, Nantong, China).

### Antibodies and Western Blot Assay

Mouse anti-GAPDH, anti-p53 and anti-Prp19 were purchased from Santa Cruz Technology (Santa Cruz, CA, USA). Rabbit anti−BCL-2, anti−MCL-1, anti−BCL-XL and cleaved poly (adenosine diphosphate-ribose) polymerase (PARP) were purchased from Cell Signaling Technology (Beverly, MA, USA).

For western blot, cells were lysed in lysis buffer (20 mM Tris-HCl, pH 7.5, 150 mM NaCl, 1 mM EDTA, and 1% Triton X-100 with protease inhibitor), and their extracts were clarified via centrifugation. The cell lysate proteins were separated on 10% SDS-PAGE gel and then transferred to polyvinylidene difluoride membrane (Millipore, Billerica, MA, USA). The membranes were blocked in blocking solution (50 mM Tris-HCl, 150 mM NaCl, 5% (w/v) non-fat dry milk and 0.1% Tween-20) at room temperature for 2 h, followed by incubation with appropriate primary antibodies at 4°C overnight. After incubating with appropriate secondary antibodies at room temperature for 1 h, gels were scanned and quantified using using ImageQuant LAS 4000 mini (GE Healthcare, NY, USA).

### Real-time reverse transcription PCR (qPCR)

Total RNA was exacted by TRIzol (Invitrogen, NY, USA). The first strand cDNA synthesis was carried out with AMV RNA PCR kit (TaKaRa, Dalian, China) according to the manufacturer's protocol. Subsequent qPCR was performed using a SYBR Green Premix Ex Taq (TaKaRa, Dalian, China) on ABI StepOne Plus system (Applied Biosystems, CA, USA). The relative mRNA level of specific genes was calculated using the ^δδ^Ct method after normalization against Glyceraldehyde-3-phosphate dehydrogenase (GAPDH). The primers of interested genes were as follows: Prp19: 5′-GTGCC AAGTT CCCAA CCAAG TGTT-3′ (forward) and 5′-AGCAC AGTGG CTTTG TCTTG AAGC-3′ (reverse), and GAPDH: 5′-TCGAC AGTCA GCCGC ATCTT CTTT-3′ (forward) and 5′-GCCCA ATACG ACCAA ATCCG TTGA-3′ (reverse).

### RNA Interference

A small interfering RNA (100 pmol/L) against Prp19 (Genepharma, Shanghai, China) was transfected into cells in 6-well plates using Lipofectamine 2000 (Invitrogen, NY, USA) according to the manufacturer's instructions. Forty-eight hours after transfection, gene silencing effects were measured by western blot. The siRNA sequences were listed in Table 1.

**Table 1:** List of siRNAs for suppressing Prp19.

siRNAs	Sequence (5′ to 3′)
siRNA1 Prp19 sense	GGCUC AUCGA GAAGU ACAUT T
siRNA1 Prp19 antisense	AUGUA CUUCU CGAUG AGCCT T
siRNA2 Prp19 sense	GCCAC UAUCA GGAUU UGGUT T
siRNA2 Prp19 antisense	ACCAA AUCCU GAUAG UGGCT T
siRNA3 Prp19 sense	GCCAA GUUCA UCGCU UCAAT T
siRNA3 Prp19 antisense	UUGAA GCGAU GAACU UGGCT T
siRNA Negative control sense	UUCUC CGAAC GUGUC ACGUTT
siRNA Negative control antisense	ACGUG ACACG UUCGG AGAAT T

### Terminal deoxynucleotidyl transferase dUTP nick end labeling (TUNEL) Assay

HCC cells were treated with CDDP or doxorubicin for 24 h. Apoptotic cells were detected with the In Situ Cell Death Detection Kit (Roche, Mannheim, Germany) according to the manufacturer' protocol, followed counterstained with 4′, 6 -diamidino-2-phenylindole. Apoptotic cells were imaged with a Nikon microscope with NIS Element F3.2 software (Nikon, Tokyo, Japan).

### Flow Cytometry Assay

Pretreated Huh7 cells were stained with FITC-AnnexinV and propidium iodide (PI), and then evaluated for apoptosis by flow cytometry according to the manufacturer's protocol (Multisciences, Hangzhou, China). Briefly, cells were harvested after the incubation period, washed twice in cold phosphate-buffered saline (PBS), and centrifuged at 2000 rpm for 5 min. About 1×10^6^ cells were re-suspended in 500 µL 1× annexin-binding buffer and transferred to a sterile flow cytometry glass tube. 5 µL FITC-AnnexinV and 10 µL PI were added to each tube, followed by incubation at room temperature for 5 min in the dark. Following incubation, samples were analyzed by flow cytometry (Epics Altra, Beckman, USA).

### Statistical analysis

Statistical analyses were conducted on SPSS 19.0 for Windows. Student's *t* test was used to compare differences between different groups. Statistical significance was set at *P*<0.05.

## Results

### Dox or CDDP induces Prp19 down-regulation in HCC cells

**Figure 1:**
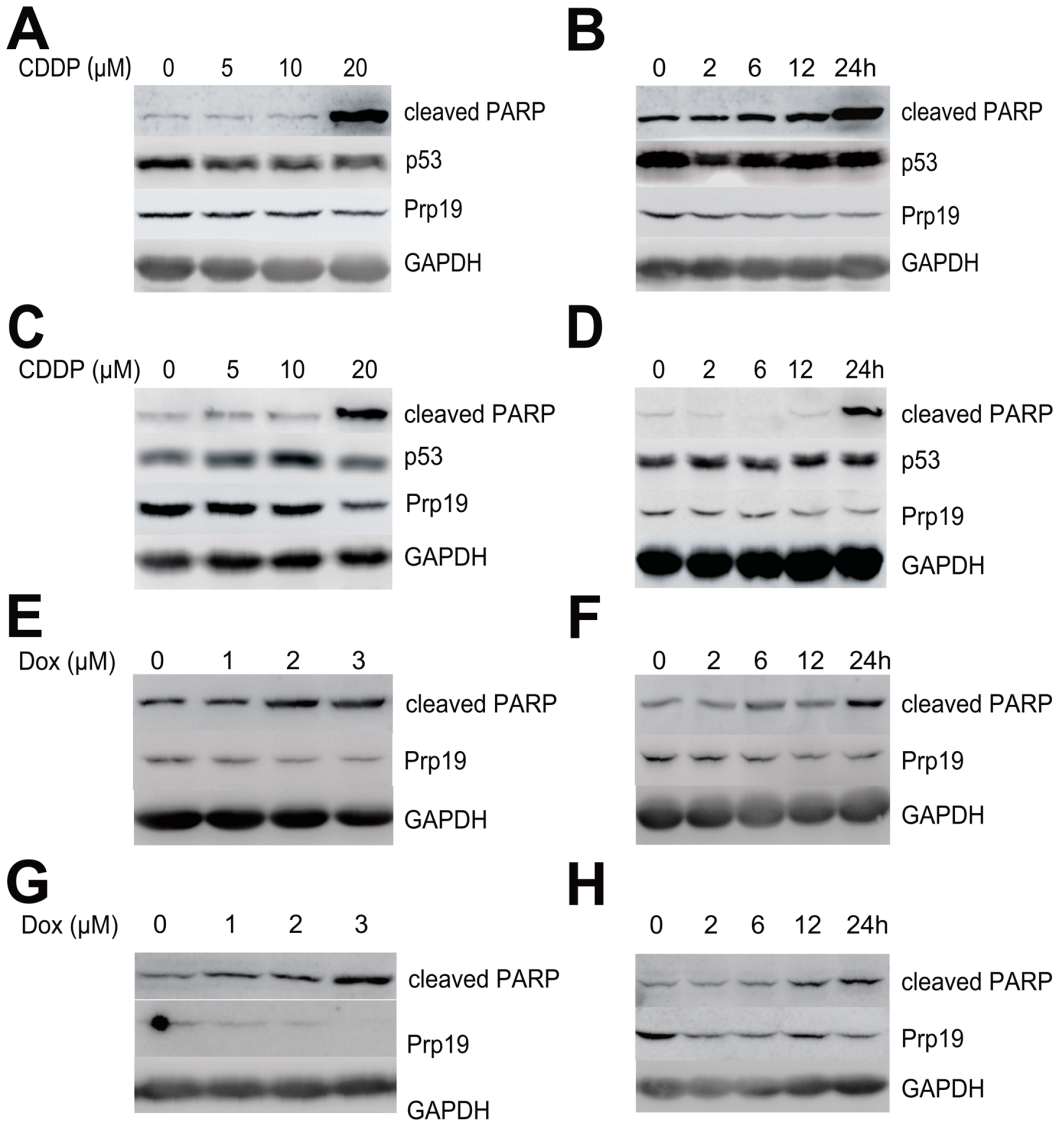
Cisplatin (CDDP) or doxorubicin (Dox) induces Prp19 down-regulation in HCC cells. (A) Huh7 cells were treated with the indicated concentration of CDDP for 24 h and cell lysates were probed with indicated antibodies. (B) Time course of 10 µM CDDP treatment in Huh7 cells. (C) SMMC-7721 cells were incubated with the indicated concentration of CDDP for 24 h. (D) Time course of 10 µM CDDP treatment in SMMC-7721 cells. (E) Huh7 cells were treated with the indicated concentration of Dox for 24 h. (F) Time course of 2 µM Dox treatment in Huh7 cells. (G) SMMC-7721 cells were treated with the indicated concentration of Dox for 24 h. (H) Time course of 2 µM Dox treatment in SMMC-7721 cells.

In contrast to normal hepatocyte L02 and liver tissue, HCC cell lines and tissue displayed greater abundance of Prp19 expression (unpublished data), suggesting that Prp19 may be involved in hepatocarcinogenesis. In normal human fibroblast, DNA crosslinking agent CDDP causes biophasic induction of Prp19, accompanied by p53 activation [14]. To investigate the response of Prp19 to DNA damage agents in HCC cells with mutated p53, Huh7 cells, expressing mutated p53 [15], was treated with CDDP at different time intervals with different doses. The results shown that the protein level of Prp19 was down-regulated by CDDP in a dose- (Fig. 1A) and time-dependent (Fig. 1B) manner, whereas p53 was not activated. In concomitant with the down-regulation of Prp19, cleaved PARP, an apoptotic marker, was increased with CDDP treatment (especially at 20 µM and 24 h) in our study. The decreased Prp19 and increased cleaved PARP by CDDP treatment can also be observed in SMMC-7721 cells, which contain wild-type p53 [16] (Fig. 1C and D). Further, Dox, a DNA double-strand break agent, also induced down-regulation of Prp19 in Huh7 cells in a dose- and time-dependent manner (Fig. 1E and F), and in SMMC-7721 cells in a dose-dependent manner (Fig. 1G) but not in a time-dependent manner (Fig. 1H). These data indicate that, DNA damage drugs can lead to Prp19 down-regulation in HCC cells in regardless of p53 status.

#### CDDP or Dox treatment compromises Prp19 stability in HCC cells

**Figure 2:**
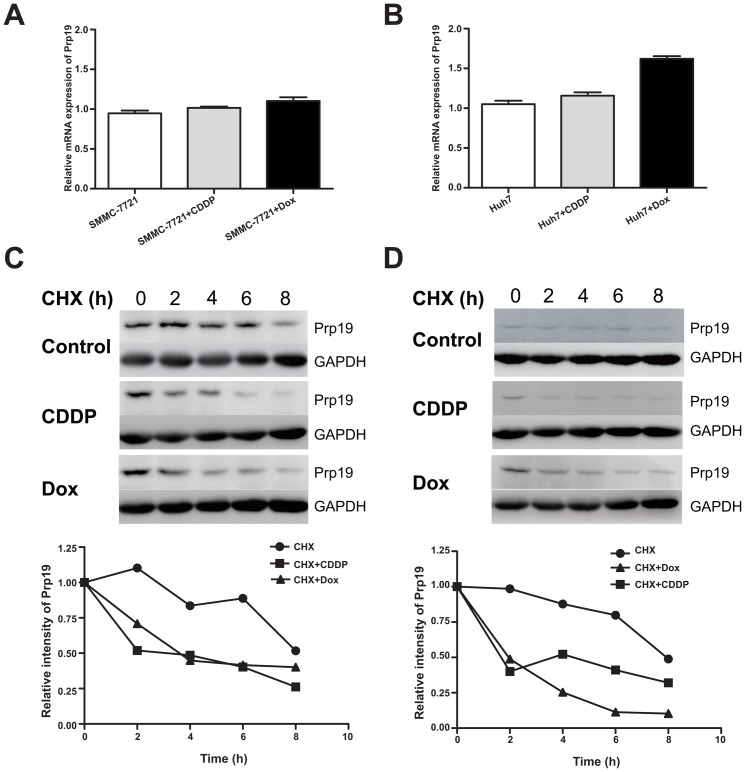
CDDP or Dox treatment compromises Prp19 stability in HCC cells. (A and B) Relative mRNA expression of Prp19 in SMMC-7721 or Huh7 cells treated with 10 µM CDDP and 2 µM Dox for 24 h. Data were presented as mean ± SD; n = 4. (C and D) SMMC-7721 cells or Huh7 cells was treated with 50 µg/mL cycloheximide (CHX) in the presence of 10 µM CDDP or 2 µM Dox at different time durations, and then Prp19 protein expression were analyzed. Densitometric values were detected and presented.

To examine whether Prp19 down-regulation was induced at transcriptional level by DNA damage agents, Prp19 mRNA level in Huh7 cells and SMMC-7721 cells was analyzed. The results showed that Prp19 mRNA level was not obviously affected by these agents in Huh7 cells (Fig. 2A), while similar results was observed in SMMC-7721 cells (Fig. 2B). These results suggest that CDDP or Dox treatment modulates Prp19 expression in HCC cells at post-transcriptional level.

It has been reported that DNA damage induces ubiquitination of Prp19 in HeLa cells [8], which is another post-transcripational modification affecting protein stability. To examine whether DNA damage agents modulated the Prp19 stability in Huh7 and SMMC-7721 cells, the stability of Prp19 was measured by treating cells with cycloheximide (CHX) in the presence of CDDP or Dox in a time-course experiment. As shown in Fig. 2C, the percentage of Prp19 protein in Huh7 cells incubated with CHX and DNA damage agents was markedly decreased as compared to incubated with CHX alone. In line with findings in Huh7 cells, CDDP or Dox treatment also impaired the stability of Prp19 in SMMC-7721 cells (Fig. 2D). These data suggest that down-regulation of Prp19 in Dox- or CDDP- treated HCC cells was mainly due to decreased stability of Prp19.

#### Silencing Prp19 enhances apoptosis of HCC cells induced by Dox or CDDP

**Figure 3:**
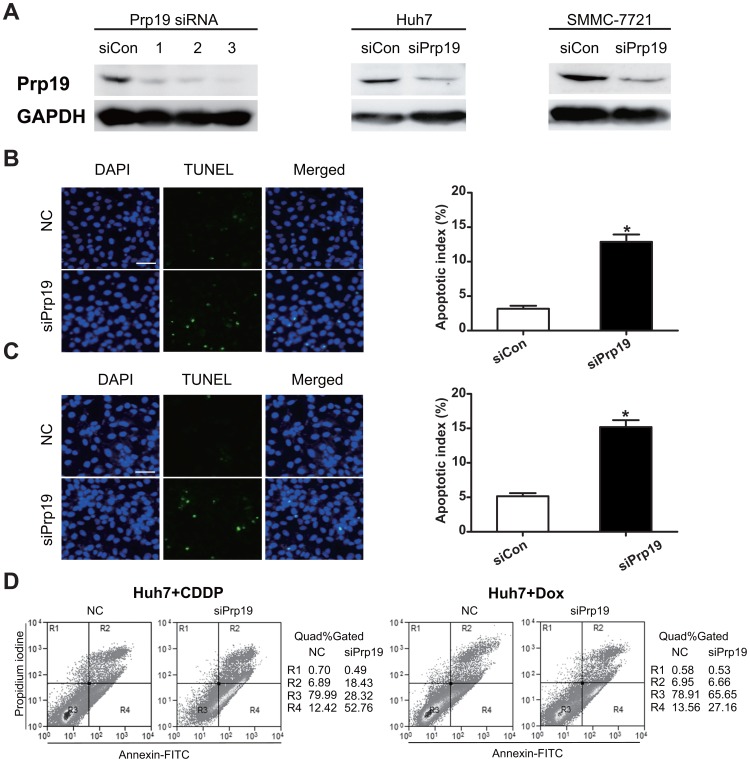
Silencing Prp19 enhances apoptosis of HCC cells induced by Dox or CDDP. (A) Huh7 cells were transfected with negative control siRNA and three siRNAs targeting Prp19, followed to assess the effect of silencing. (B and C) Huh7 or SMMC-7721 cells were transfected with negative control siRNA and Prp19 siRNA2 for 48 h, and incubated with 10 µM CDDP for another 24 h. Apoptosis was detected by in situ nick end-labeling of fragmented DNA with terminal deoxynucleotidyl transferase assay. The apoptotic index, defined as the percentage of apoptotic cells, was calculated and summarized in the bar chart. The values represent the mean standard deviation of 3 independent experiments (Scale bar, 50 µm; *P<0.001). (D) Huh7 cells were transfected with negative control siRNA and Prp19 siRNA2 for 48 h, and incubated with 10 µM CDDP (left) or 2 µM Dox (right) for another 24 h. Apoptosis was determined by flow cytometry. Cells stained with Annexin-V–fluorescein isothiocyanate were counted as apoptotic, and the apoptotic index was defined as the percentage of apoptotic cells.

Next, to evaluate the effect of Prp19 on apoptosis of HCC cells induced by DNA damage agents, Prp19 protein expression was repressed using siRNA against Prp19 and then incubated with DNA damage agents. Finally apoptosis was detected by TUNEL assay and flow cytometry after staining with Annexin V and PI. In contrast to negative control siRNA, Prp19 siRNA3 efficiently inhibited Prp19 expression in these two cell lines, and was then used in following transient transfection experiments (Fig. 3A). TUNEL assay revealed that knockdown Prp19 significantly potentiated CDDP-induced apoptosis in Huh7 and SMMC -7721 cells (Fig. 3B and C). Similar to TUNEL assay results, flow cytometry histogram showed that repressing Prp19 in Huh7 cells enhanced CDDP- or Dox- induced apoptosis (Fig. 3D). These results provide evidence that depletion of Prp19 in HCC cells render them more sensitive to DNA damage agents-induced apoptosis.

#### Down-regulation of Prp19 decreases pro-survival proteins expression and increases PARP cleavage in HCC cells

**Figure 4:**
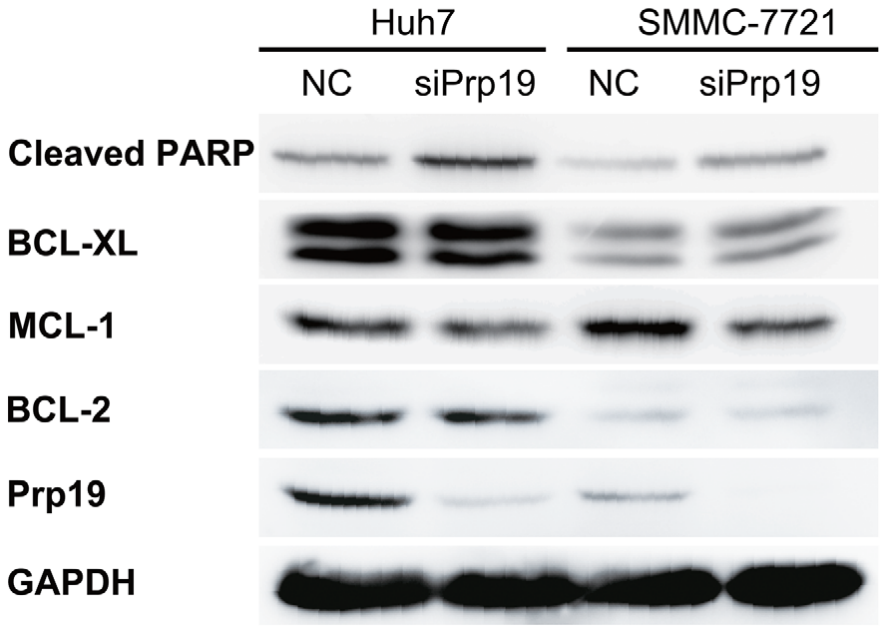
Down-regulation of Prp19 decreases pro-survival proteins expression and increases PARP cleavage in HCC cells. Huh7 and SMMC-7721 cells were transfected with indicated siRNAs.

To explore the underlying mechanisms, we measured the expression of pro-survival proteins in Prp19-defective HCC cells. Transient knockdown Prp19 in both Huh7 and SMMC-7721 cells down-regulated MCL-1 expression, while BCL-XL and BCL-2 levels were not affected (Fig. 4). Moreover, increased cleaved PARP was also observed after repressing endogenous Prp19 expression in HCC cells (Fig. 4), indicating that depletion of Prp19 resulted in increased basal apoptosis. These observations indicate that Prp19 may implement its regulation on apoptosis of HCC cells by affecting expression of anti-apoptotic proteins MCL-1.

## Discussion

Although patients with unresectable HCC benefits from chemoembolization with Dox or CDDP, benefit of this adjuvant modality for HCC remains less investigated [17]. Resistance to Dox or CDDP induced-apoptosis partially contributes to this therapeutic issue [18]. In cancer cells with functional p53, DNA damage triggers activation of p53 and its target genes, resulting in cell cycle arrest and apoptosis [19]. In the absence of p53, DNA damage can also induce apoptosis in cancer cells via other mechanisms [20], [21].

Previous studies have revealed that Prp19 plays diverse role in DNA damage repair, resulting in decreased cell apoptosis and enhanced cell survival under DNA damage [9], [14]. Prp19 alone or in combination with its complex can interact with DNA repair proteins such as terminal deoxynucleotidyl transferase and Metnase to implement damage repairing and suppress apoptosis [14], [22]. Furthermore, Prp19 interacts with PHD3 to lessen cell death under hypoxic conditions [23]. In response to several DNA damage agents, biphasic induction of Prp19 is observed in normal human fibroblast to execute its pro-survival function [14]. Here we found that upon DNA damage, Prp19 was down-regulated in a time- and dose-dependent manner in HCC cells with mutated or wild-type p53. Silencing Prp19 enhanced CDDP or Dox-induced apoptosis in these two HCC cells, which was in line with previous findings in HeLa cells [8].

Direct involvement of Prp19 in DNA damage response has been reported in several studies. Prp19 is ubiquitinated in HeLa cells under DNA damage, leading to enhanced stability of other components within Prp19-associated complex[8]. Recently, ATM dependent phosphorylation of Prp19 upon DNA damage or oxidative stress is identified to be necessary for mediating the resistance to apoptosis upon oxidative stress and elongating the cellular life span [9]. Since both DNA damage and Prp19 overexpression can trigger self-ubiquitination [8], the fall back after the peak of Prp19 induction may be due to increased self-ubiquitination and subsequent impaired stability [14]. Our current study also displayed DNA damage-mediated down-regulation of Prp19 in HCC cells was mainly attributed to impairment in Prp19 stability. Hence, more studies are required to answer whether DNA damage-mediated down-regulation of Prp19 is dependent on ubiquitin/proteasome.

In addition, exogenous Prp19 lowers basal DNA damage and apoptosis, elongating cellular life span [9]. Here we also demonstrated that knockdown endogenous Prp19 enhanced cleaved PARP without extracellular stress, which indirectly supported its pro-survival role in both normal human cells and cancer cells. Furthermore, we found that Prp19 modulated expression of pro-survival proteins MCL-1, which may be helpful to elucidate the function of Prp19 in HCC cells under DNA damage.

In summary, our work, for the first time, demonstrated that DNA damage impaired Prp19 stability and resulted in Prp19 down-regulation. Knockdown of Prp19 in HCC cells potentiated the pro-apoptotic effect of chemotherapeutic drugs, suggesting that targeting Prp19, together with chemoembolization, can make this treatment more efficient. Moreover, investigating the mechanism for Prp19-mediated expression of MCL-1 may provide more insights into chemotherapeutic resistance in HCC.
